# NONHSAT076754 aids ultrasonography in predicting lymph node metastasis and promotes migration and invasion of papillary thyroid cancer cells

**DOI:** 10.18632/oncotarget.13725

**Published:** 2016-11-30

**Authors:** Shujun Xia, Chuandong Wang, Xiaofeng Ni, Zhongxin Ni, Yijie Dong, Weiwei Zhan

**Affiliations:** ^1^ Ultrasound Department, Rui Jin Hospital Shanghai Jiao Tong University School of Medicine, Shanghai, P. R. of China; ^2^ The Key Laboratory of Stem Cell Biology, Institute of Health Sciences, Shanghai Jiao Tong University School of Medicine & Shanghai Institutes for Biological Sciences, Chinese Academy of Sciences, Shanghai, P. R. of China

**Keywords:** NONHSAT076754, papillary thyroid cancer, lymph node metastasis, ultrasonography, biomarker

## Abstract

Lymph node metastasis (LNM) is the primary challenge in papillary thyroid cancer (PTC). Recurrent cancerous lymph nodes require repeated surgeries, which increases the risk for surgical complications. Thus, the evaluation of LNM before surgery is important. Ultrasonography is the most convenient way to examine cervical LNM, but the sensitivity of ultrasonography in the identification of LNM in cases of PTC is extremely low. A series of lncRNAs (long noncoding RNAs) have been reported as candidate biomarkers in a variety of tumors. This study detected the lncRNA NONHSAT076754 in PTC and analyzed the correlation of NONHSAT076754 with the clinicopathological and ultrasonographic characteristics of patients with PTC. The value of NONHSAT076754 as an auxiliary diagnostic biomarker for use along with ultrasonography in the differentiation of LNM in PTC was assessed. Additionally, the biological function of NONHSAT076754 in PTC cells was demonstrated. Our study indicated that NONHSAT076754 promotes migration and invasiveness of PTC and serves as a valuable auxiliary biomarker that can be used along with ultrasonography in the prediction of cervical LNM.

## INTRODUCTION

Thyroid cancer is recognized as a common endocrine malignancy. Its incidence has been rapidly increasing in recent years because of more accurate ultrasonographic technology and the application of ultrasound-guided fine-needle aspiration (US-FNA) [1–4]. Papillary thyroid cancer (PTC), which accounts for the majority of cases of thyroid cancer with a good prognosis, is most likely to result in regional lymph node recurrence [5, 6]. Even though recurrent lymph node metastasis (LNM) is not lethal, it is one of the major challenges in the management of patients with PTC [7]. Repetitive surgeries may increase the risk of surgical complications, and radioiodine ablation may not be effective since some patients do not exhibit sufficient radioiodine uptake or they demonstrate positive metastasis by 18F-fluorodeoxyglucose positron emission tomography (18FDG-PET) [8, 9]. LNM in PTC is not only an indicator of recurrence but is also used as a reference for the surgical management of patients before surgery [10]. Detection by ultrasound is the most convenient way to examine LNM in PTC, but its sensitivity remains extremely low [11, 12].

Long noncoding RNAs (lncRNAs), which comprise a class of long RNAs (more than 200 nucleotides) without protein coding potential, are involved in various biological processes, including cell proliferation, differentiation, apoptosis, development and immune responses [13, 14]. Some recent reports have demonstrated that lncRNAs play a critical role in cancer development and progression and that they have the potential to serve as diagnostic and prognostic biomarkers in gastric cancer [15], prostate cancer [16], and hepatocellular carcinoma [17], among other cancers. LncRNAs are tumor-specific and are also very stable in serum, urine and other easily obtainable body fluids [18], which indicates that they are good candidate tumor biomarkers. However, conclusions about the diagnostic value of lncRNAs in thyroid cancer are lacking.

The lncRNA NONHSAT076754 is a transcript of fibronectin 1, which is a cell surface and extracellular protein that is involved in cell adhesion and migration processes including those of wound healing [19], blood coagulation [20], and metastasis [21], among others. The present study investigated the expression of NONHSAT076754 in PTC and its correlation with patient characteristics. The diagnostic value of NONHSAT076754 and ultrasonography in the identification of LNM in PTC was analyzed in clinical samples. Moreover, the biological function of NONHSAT076754 was assessed in PTC cell lines. This study aims to identify NONHSAT076754 as an auxiliary biomarker for use with ultrasonography in the prediction of LNM and its role in PTC.

## RESULTS

### Patient demographics

As shown in Table 1, 72 patients were included in our study. They were classified into two groups (LNM group, n = 37; nonLNM group, n = 35) according to whether the patients had LNM. The distribution of sex, age and multifocality in the two groups was the same (P >0.05). Among all these patients, only one (1.4%) patient who was in the LNM group exhibited extrathyroid extension. According to the American Joint Committee on Cancer (AJCC) TNM staging system (7th edition), the tumors were all designated pT1, and no patients had distant metastasis. In the LNM group, 64.9% of the patients were N1a and 35.1% were N1b. Patients with LNM were more likely to be in an advanced stage of the disease (III/IV) (P < 0.05).

**Table 1:** Patient demographics

Clinicopathological characteristics	All patients, *n* (%)	LNM(*n* = 37)	nonLNM(*n* = 35)	*P* value
Sex				0.900
Male	16(22.2%)	8(21.6%)	8(22.9%)	
Female	56(77.8%)	29(78.4%)	27(77.1%)	
Age(mean±SD)		45.70±13.25	46.14±10.66	0.877
<45 y	31(43.1%)	16(43.2%)	15(42.9%)	
≥45 y	41(56.9%)	21(56.8%)	20(57.1%)	
Extrathyroid extension				0.327
Presence	1(1.4%)	1(2.7%)	0(0.0%)	
Absence	71(98.6%)	36(97.3%)	35(100.0%)	
Multifocality				0.071
Unifocal	53(73.6%)	24(64.9%)	29(82.9%)	
Multifocal	19(26.4%)	13(35.1%)	6(17.1%)	
Tumor(T)				0.974
T1a	35(48.6%)	15(40.5%)	20(57.1%)	
T1b	37(56.4%)	22(59.5%)	15(42.9%)	
Lymph node(N)				/
N0	35(48.6%)	/	/	
N1a	24(33.3%)	/	/	
N1b	13(18.1%)	/	/	
Metastasis(M)				
M0	72(100%)	/	/	/
pTNM stage				
Early(I/II)	50(69.4%)	15(40.5%)	35(100.0%)	0.000*
Advanced(III/IV)	22(30.6%)	22(59.5%)	0(0.0%)	

* P < 0.05; LNM = lymph node metastasis

### NONHSAT076754 overexpression is associated with LNM in PTC

**Figure 1:**
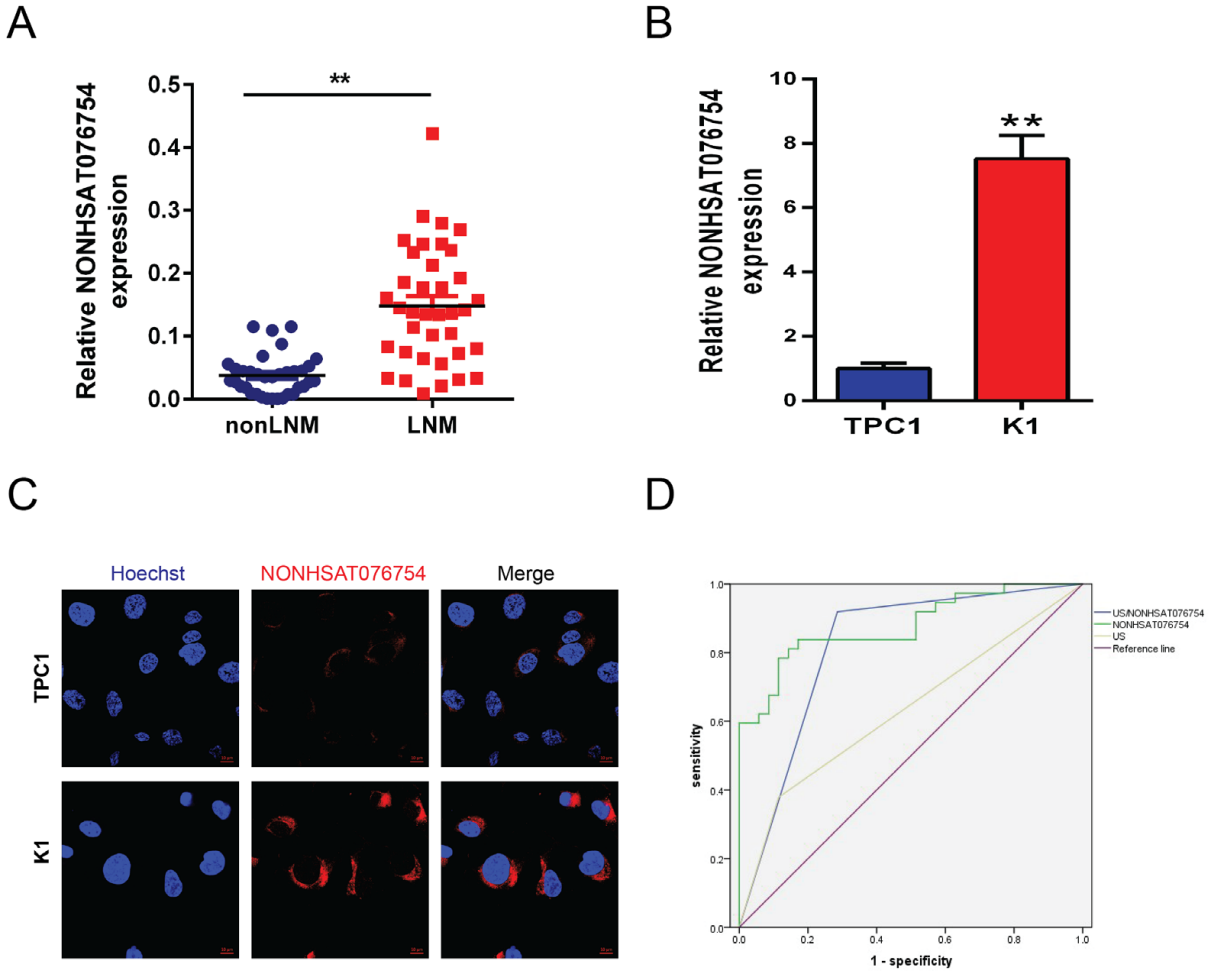
LncRNA NONHSAT076754 is overexpressed in PTC with LNM The expression level of NONHSAT076754 was detected in PTC tissues (nonLNM (n = 35) and LNM (n = 37)) A. and cell lines B. by qRT-PCR. The value was normalized to GAPDH mRNA expression. “**” indicates P < 0.05. C. FISH analysis of the subcellular distribution of NONHSAT076754 in TPC1 and K1 cells as detected by confocal microscopy. Nuclei (blue) were labeled with Hoechst solution; the NONHSAT076754 probe (red) was labeled with Cy3. Representative images of three repeated experiments are shown. The scale bar is 10 μm. D. The ROC curves of ultrasonography and NONHSAT076754 predicted potential diagnostic values of ultrasonography and NONHSAT076754. NONHSAT076754: AUC 0.878 (0.798, 0.958), P <0.01; US/NONHSAT076754: AUC 0.817 (0.712, 0.921), P <0.01.

The expression level of NONHSAT076754 was detected in tumor tissues of the 72 patients. NONHSAT076754 was significantly up-regulated in 37 metastatic PTCs compared with 35 nonmetastatic PTCs (Figure 1A). We further analyzed the correlation of NONHSAT076754 expression and the clinicopathological and ultrasonographic characteristics of all these patients. Furthermore, the expression level of NONHSAT076754 was determined by a receiver operating characteristic (ROC) curve. The cutoff value of NONHSAT076754 expression was calculated by ROC curve analysis. The highest diagnostic value was at a cutoff of △Ct = 4.152, according to which all PTC patients were categorized into either the low-expression group (n = 35, △Ct > 4.152) or the high-expression group (n = 37, △Ct < 4.152). As summarized in Table 2, NONHSAT076754 overexpression was significantly correlated with LNM in PTC (N0 vs N1a&N1b, P = 0.009) as well as with pTNM stage (I-II vs III-IV, P = 0.022). However, no significant associations were observed between NONHSAT076754 expression and sex (P = 0.576), age (P = 0.476), extrathyroid extension (P = 1.000), multifocality (P = 1.000) or tumor stage (P = 0.098). For the ultrasonographic characteristics, no significant differences were observed between the two groups (Table 3). Moreover, the univariate and multivariate logistic regression analyses showed that NONHSAT076754 overexpression was a strong independent diagnostic factor for LNM in patients with PTC (P = 0.000) (Table 4).

**Table 2:** Correlations between NONHSAT076754 expression and ultrasonographic characteristics of PTC

Clinicopathological characteristics	*n*	High expression	Low expression	*P* value
Sex				0.576
Male	16	7(18.9%)	9(25.7%)	
Female	56	30(81.1)	26(74.3%)	
Age				
<45y	31	14(37.8%)	17(48.6%)	0.476
≥45y	41	23(62.2%)	18(51.4%)	
Extrathyroid extension				1.000
Presence	1	1(2.7%)	0(0.0%)	
Absence	71	36(97.3%)	35(100%)	
Multifocality				1.000
Unifocal	53	27(73.0%)	26(74.3%)	
Multifocal	19	10(27.0%)	9(25.7%)	
Tumor(T)				0.098
T1a	35	14(37.8%)	21(60.0%)	
T1b	37	23(62.2%)	14(40.0%)	
Lymph node(N)				0.009*
N0	35	12(32.4%)	23(65.7%)	
N1a	24	18(48.6%)	6(17.1%)	
N1b	13	7(18.9%)	6(17.1%)	
Metastasis(M)				/
M0	72(100%)	/	/	
pTNM stage				0.022*
Early(I/II)	50	21(56.8%)	29(82.9%)	
Advanced(III/IV)	22	16(43.2%)	6(17.1%)	

**Table 3:** Correlations between NONHSAT076754 expression and ultrasonographic characteristics of PTC

Ultrasonographic characteristics	*n*	High expression	Low expression	*P* value
Border				0.788
Clear	18	10(27.0%)	8(22.9%)	
Obscure	54	27(73.0%)	27(77.1%)	
Margin				0.795
Regular	20	11(29.7%)	9(25.7%)	
Irregular	52	26(70.3%)	26(74.3%)	
Shape				1.000
Taller than wide	6	3(8.1%)	3(8.6%)	
Wider than tall	66	34(91.9%)	32(91.4%)	
Halo				0.540
Present	7	4(10.8%)	3(8.6%)	
Absent	65	33(89.2%)	32(91.4%)	
Internal architecture				0.240
Solid	69	34(91.9%)	35(100.0%)	
Mostly solid	3	3(8.1%)	0(0.0%)	
Echogenicity				0.984
Markedly hypoechoic	18	9(24.3%)	9(25.7%)	
Hypoechoic	46	24(64.9%)	22(62.9%)	
Isoechoic	8	4(10.8%)	4(11.4%)	
Echo homogeneity				0.460
Homogenous	24	14(37.8%)	10(28.6%)	
Inhomogeneous	48	23(62.2%)	25(71.4%)	
Calcification				0.329
None	31	14(37.8%)	17(48.6%)	
Micro	33	20(54.1%)	13(37.1%)	
Macro	8	3(8.1%)	5(14.3%)	
Contact				0.123
0-25%	57	27(73.0%)	30(85.7%)	
25-50%	11	6(16.2%)	5(14.3%)	
>50%	4	4(10.8%)	0	
Lateral shadow				0.486
Absence	71	37(100.0%)	34(97.1%)	
Presence	1	0	1(2.9%)	
Blood flow				0.411
None	25	10(27.0%)	15(42.9%)	
Low	23	12(32.4%)	11(31.4%)	
Medium	14	8(21.6%)	6(17.1%)	
High	10	7(18.9%)	3(8.6%)	
Vascularity				0.412
None	24	11(29.7%)	13(37.1%)	
Circular	19	8(21.6%)	11(31.4%)	
Central	1	1(2.7%)	0(0.0%)	
Mix	28	17(45.9%)	11(31.4%)	

*P < 0.05

**Table 4:** Univariate and multivariate regression analyses of parameters associated with LNM

Parameters	Category	Univariate analysis		Multivariate analysis	
		OR(95% CI)	*P* value	OR(95% CI)	*P* value
Sex	Male/Female	0.484 (0.155-1.515)	0.212		
Age	<45/≥45y	1.016 (0.399-2.583)	0.974		
Multifocality	Unifocal/Multifocal	0.700 (0.243-2.017)	0.509		
Tumor size	T1a/T1b	0.511 (0.200-1.306)	0.161		
Stage	Early/Advanced	1.136 (1.04-1.464)	0.001	1.149 (1.031-1.703)	0.016*
NONHSAT076754	Low/High	1.040 (1.012-1.138)	0.000	1.042 (1.011-1.160)	0.000*

* P < 0.05; LNM = lymph node metastasis; OR = odds ratio; CI = confidence interval

### Combined diagnostic value of NONHSAT076754 with ultrasonography of LNM in PTC

To further evaluate the possibility of the clinical application of NONHSAT076754 in patients with PTC, the diagnostic potential and discriminatory accuracy of ultrasonography-NONHSAT076754 was evaluated by ROC curve analysis and the corresponding area under the curve (AUC) values. The AUC for NONHSAT076754 was 0.878 (95% confidence interval (CI) = 0.798-0.958; P < 0.001, Figure 1D). The level of NONHSAT076754 overexpression showed a sensitivity of 83.78% and a specificity of 82.85% with a diagnostic accuracy of 83.33%, whereas the sensitivity and specificity of ultrasonography were 35.14% and 88.57%, respectively, with a diagnostic accuracy of 61.11%. The combined diagnostic value of ultrasonography-NONHSAT076754 was increased; the sensitivity was 91.89%, the specificity was 82.85%, and the accuracy was 87.50%. (Table 5)

**Table 5:** Performance of US-NONHSAT076754 in the differentiation of LNM from nonLNM in PTC

	LNM	Sensitivity	Specificity	NPV	PPV	Accuracy
US	+ 17	35.14%	88.57%	56.36%	76.47%	61.11%
	-55	(13/37)	(31/35)	(31/55)	(13/17)	(44/72)
NONHSAT076754	+37	83.78%	82.85%	82.85%	83.78%	83.33%
	-35	(31/37)	(29/35)	(29/35)	(31/37)	(60/72)
US-NONHSAT076754	+40	91.89%	82.85%	90.62%	85.00%	87.50%
	-32	(34/37)	(29/35)	(29/32)	(34/40)	(63/72)

US = ultrasonography; LNM = lymph node metastasis; NPV = negative predictive value; PPV = positive predictive value

### The expression and subcellular distribution of NONHSAT076754 in PTC cell lines

The expression of NONHSAT076754 was further confirmed in two PTC cell lines (TPC1, K1) by reverse transcription-quantitative polymerase chain reaction (RT-qPCR). The results showed that the expression of NONHSAT076754 was significantly higher in the K1 cell line than in the TPC1 cell line (Figure 1B). The results obtained in cell lines were consistent with those obtained in patient samples. Additionally, the fluorescence in situ hybridization (FISH) analysis demonstrated that both TPC1 and K1 cells exhibited positivity in the cytoplasm when a fluorescence-conjugated NONHSAT076754 probe was used, which shows that NONHSAT076754 is a lncRNA that is distributed in the cytoplasm. The fluorescent signal in K1 cells was much higher than that in TPC1 cells, which also indicates that the expression level of NONHSAT076754 in K1 cells was higher than that in TPC1 cells. (Figure 1C)

### Overexpression of NONHSAT076754 promotes the migration and invasiveness of TPC1 cells

**Figure 2:**
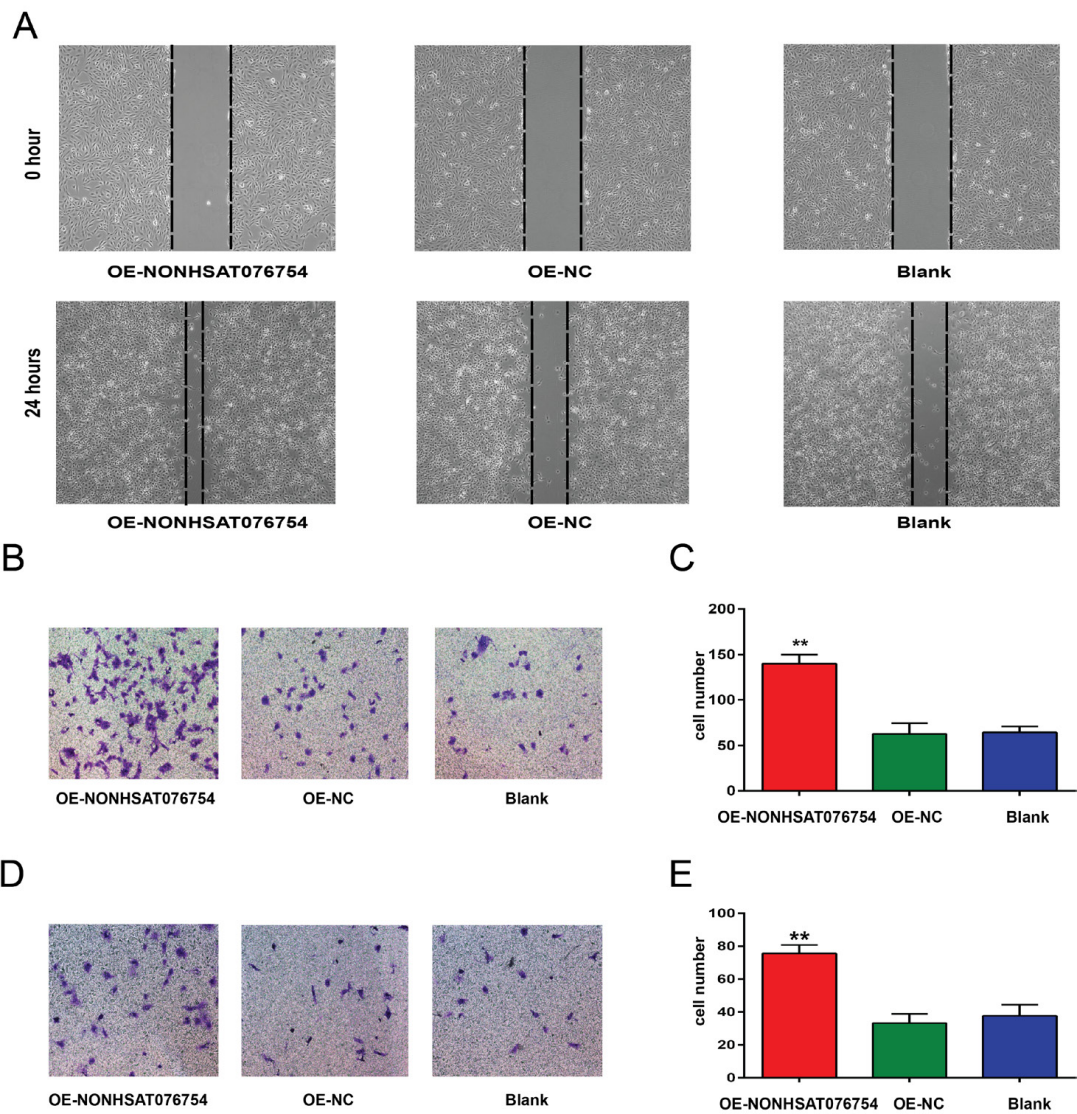
Effect of NONHSAT076754 on the migration and invasiveness of TPC1 cells **A.** A scratch test was performed to determine the migration ability of TPC1 cells. Representative images at 0 and 24 hours from three repeated experiments are shown. **B.** A Transwell assay was performed to determine the migration ability of TPC1 cells. Representative images show invasive cells in the lower chamber stained with crystal violet. **C.** The quantification of cell migration is presented as the number of migrating cells. Data are expressed as the means±SD of three independent experiments. **D.** A Transwell assay was performed to determine the invasion ability of TPC1 cells. Representative images show invasive cells in the lower chamber stained with crystal violet. **E.** The quantification of cell invasion is presented as number of migrating cells. Data are expressed as the means±SD of three independent experiments. “**” indicates P < 0.01.

A scratch assay was performed to examine the migration ability of TPC1 cells after NONHSAT076754 was overexpressed (OE-NONHSAT076754 group). We prepared stable NONHSAT076754-overexpressing transfectants using a lentiviral system (see supplementary Figure S1A). After transfection, TPC1 cells were plated in a 6-well plate. When the cells reached 100% confluence, 1-mL sterile pipet tips were used to scratch the cells. After a 24 h incubation, the distance of the scratch wound in the OE-NONHSAT076754 group was found to be significantly smaller compared with that in the control group (Figure 2A). To further detect the effect of NONHSAT076754 on migration and invasion, a transwell assay was performed in TPC1 cells. The number of cells in the OE-NONHSAT076754 group that migrated through the chamber (140±10) was significantly higher than the number of cells in the OE-negative control (NC) group (62.67±11.68) and the Blank group (64.33±6.66) (Figure 2B, 2C). A similar result was also shown in an invasion assay (Figure 2D, 2E): OE-NONHSAT076754 group: 75.67±5.13, OE-NC group: 33.33±5.51, Blank group: 37.67±6.81. The data indicate that NONHSAT076754 promotes the migration and invasiveness of PTC cells.

### Knockdown of NONHSAT076754 inhibits the migration and invasiveness of K1 cells

**Figure 3:**
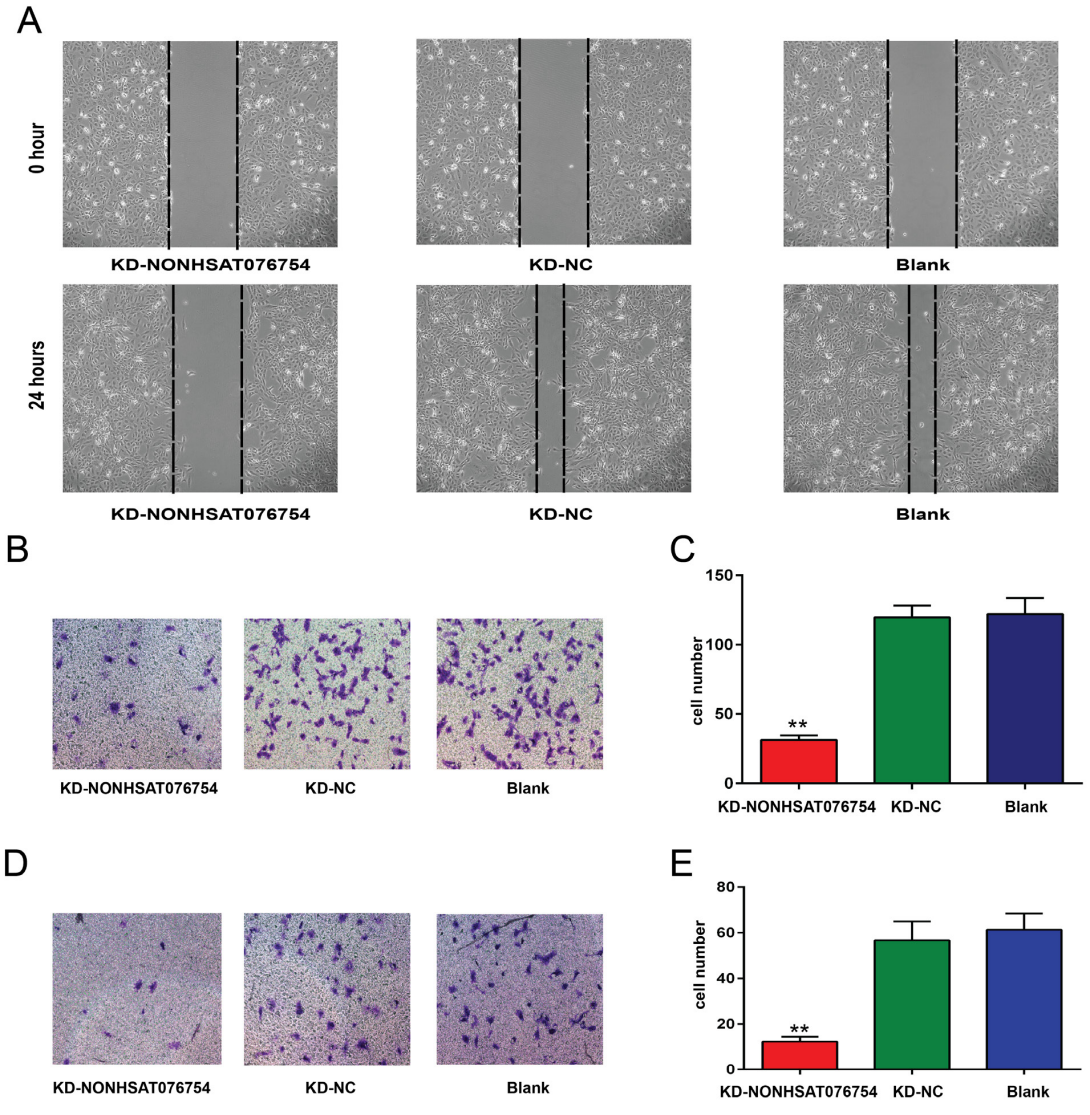
Effect of NONHSAT076754 on the migration and invasiveness of K1 cells **A.** A scratch test was performed to determine the migration ability of K1 cells. Representative images at 0 and 24 hours from three repeated experiments are shown. **B.** A Transwell assay was performed to determine the migration ability of K1 cells. Representative images show invasive cells in the lower chamber stained with crystal violet. **C.** The quantification of cell migration is presented as the number of migrating cells. Data are expressed as the means±SD of three independent experiments. **D.** A Transwell assay was performed to determine the invasion ability of K1 cells. Representative images show invasive cells in the lower chamber stained with crystal violet. **E.** The quantification of cell invasion is presented as the number of migrating cells. Data are expressed as the means±SD of three independent experiments. “**” indicates P < 0.01.

Transient knockdown of NONHSAT076754 expression was achieved using antisense oligonucleotides (see supplementary Figure S1B). Scratch and Transwell assays were also performed. K1 cells were plated in a 6-well plate and were cells were scratched by a 1-mL sterile pipet tip when they reached 100% confluence. After a 24-h incubation, the distance of the scratch wound in the KD-NONHSAT076754 group was significantly larger than that in the control group (Figure 3A). Moreover, a Transwell assay was also performed in K1 cells. The number of KD-NONHSAT076754 cells that migrated through the chamber (31.33±3.21) was significantly lower than the number of KD-NC cells (119.67±8.50) and cells in the Blank group (122±11.53) (Figure 3B, 3C). A similar result was also obtained in the invasion assay (Figure 3D, 3E): KD-NONHSAT076754 group: 12.33±2.08, KD-NC group: 50.67±8.33, Blank group: 61.33±7.09. The data indicate that NONHSAT076754 inhibits the migration and invasiveness of PTC cells.

### Effect of NONHSAT076754 on apoptosis, the cell cycle and cell proliferation in PTC

**Figure 4:**
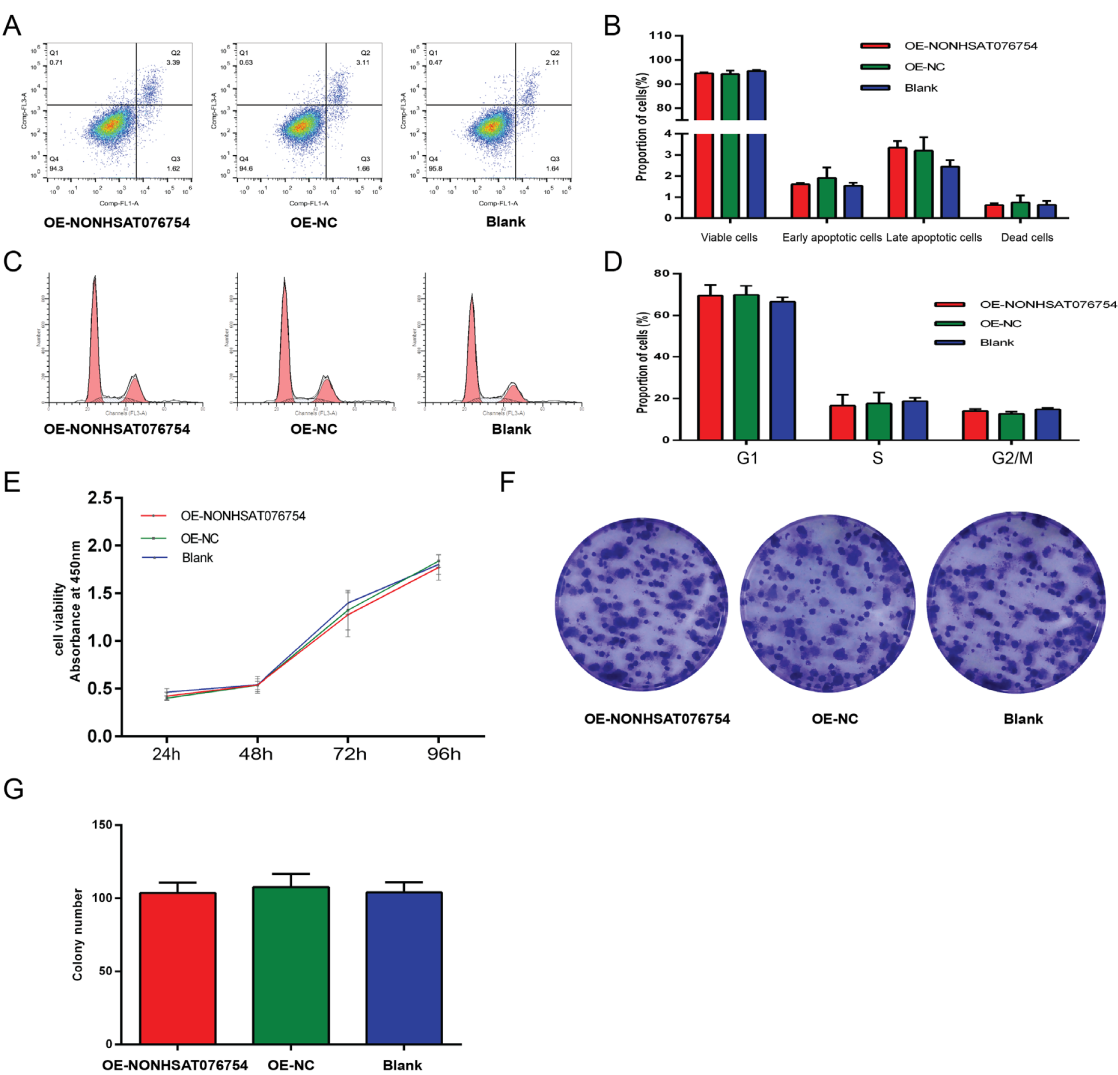
Effect of NONHSAT076754 on the proliferation and cell cycle status of TPC1 cells **A.** Images from the flow cytometric analysis of apoptosis in TPC1 cells. **B.** The percentage of apoptotic cells is presented in the histogram. Data are expressed as the means±SD of three independent experiments. **C.** Images from the flow cytometric analysis of the cell cycle in TPC1 cells. **D.** Results quantified in the cell cycle analysis are shown as a percentage of the total number of cells. Data are expressed as the means±SD of three independent experiments. **E.** A CCK-8 assay was performed to determine the proliferation of TPC1 cells transfected with NONHSAT076754 after 24 hours, 48 hours, 72 hours and 96 hours. **F.** A colony formation assay was performed to determine the proliferation of TPC1 cells transfected with NONHSAT076754. The colonies were captured and counted. **G.** The colony formation assay results are presented in the histogram. All experiments were performed independently in triplicate, and the results are presented as the means±SD.

Flow cytometry, which was used to detect the level of apoptosis, and cell cycle analysis were performed to detect the influence of NONHSAT076754 on the apoptosis and the cell cycle status of PTC cells. According to the flow cytometric apoptosis analysis, signals from apoptotic cells and viable cells were similar in the OE-NONHSAT076754 group and the control groups (Figure 4A, 4B). This result was consistent between the KD-NONHSAT076754 group and the control groups (see supplementary Figure S2A, S2B). Cell cycle analysis showed that the proportion of cells in the OE-NONHSAT076754 group that were in G1, S, and G2/M phases were similar to those in the OE-NC and Blank groups (Figure 4C, 4D). Similarly, no difference was observed in the proportion of cells in the KD-NONHSAT076754 group, the KD-NC group and the Blank group in each phase (see supplementary Figure S2C, S2D).

In the CCK8 assay, the proliferation abilities of TPC1 and K1 cells were examined at four time points (24 h, 48 h, 72 h, 96 h) after transfection. The cell viabilities are shown in a proliferation curve, which was generated based on the absorbance at 450 nm. The proliferation ability of cells in the OE-NONHSAT076754 group was similar to that in the control groups (Figure 4E). The same variation trend was found in K1 cells (see supplementary Figure S2E). In addition, the impact of NONHSAT076754 on the proliferation of TPC1 and K1 cells was determined by colony formation assay. In the colony formation assay, the number of clones was not different between the OE-NONHSAT076754 group and the control groups (Figure 4F, 4G) and a similar variation was found in K1 cells (see supplementary Figure S2F, S2G). The results indicate that apoptosis, the cell cycle and proliferation ability are not affected by NONHSAT076754.

## DISCUSSION

In this study, NONHSAT076754 was significantly increased in PTC patients with LNM compared with PTC patients without LNM. Our study indicated for the first time that increased NONHSAT076754 expression is associated with LNM of PTC and advanced pTNM stage. Our study also analyzed the diagnostic value of ultrasonography and NONHSAT076754 in the differentiation of LNM in PTC. The sensitivity of ultrasonography was very low, while the specificity was high, which was consistent with previous reports [22]. The sensitivity was highly increased when NONHSAT076754 and ultrasonography were combined for diagnosis, which allowed the diagnostic value to increase substantially. Moreover, the in vitro function study demonstrated that NONHSAT076754 promotes the migration and invasiveness of PTC cells. This study, for the first time, identified NONHSAT076754 as a valuable clinical biomarker due to its ability to complement ultrasonography in the prediction of LNM in PTC.

LNM in PTC is not fatal, but the presence of LNM would definitely determine whether the surgeon should perform lymph node dissection or recommend I131 therapy and assists in the prediction of the recurrence and prognosis of patients with PTC. Thus, it is important to obtain information on lymph node status before surgery is performed. Ultrasound imaging, as the primary method used for the preoperative assessment of cervical lymph nodes, has a low sensitivity. The way in which cervical lymph node status is evaluated needs to be optimized. Tumor biomarkers are a useful tool in the diagnosis and postoperative surveillance in various cancers [23–27]. However, biomarkers of LNM in PTC have never been reported. This is the first study that presents a LNM- associated biomarker for PTC.

The emerging field of lncRNAs has indicated their clinical potential with respect to a better understanding and management of different types of tumors. It was reported in previous studies that specific lncRNAs might be potent prognostic and predictive biomarkers [28, 29]. LncRNAs do have some advantages as clinical biomarkers. First, lncRNAs are considered more tissue-specific than protein-coding genes. It was found that the lncRNA PCA3 in urine has a higher specificity and sensitivity than serum PSA in prostate cancer patients [16, 30]. Second, circulating RNA, which is mostly derived from tumor secretions, remains quite stable under extreme conditions in the blood, despite low pH and the presence of enzymes, because of the protection afforded by membranous vesicles [18, 31]. LncRNA is an applicable biomarker in blood samples, which are easy to obtain before surgery. Furthermore, serum lncRNA is able to be tested by traditional PCR, which is the most convenient and simple laboratory technology available [17].

This study investigated the clinical relevance of NONHSAT076754 in PTC. Our study confirmed the correlation of NONHSAT076754 with LNM in PTC. NONHSAT076754 is positively associated with LNM in PTC. When combined with the detection of NONHSAT076754, the diagnostic value of ultrasonography was also greatly increased for the differentiation of PTC with LNM from PTC without LNM. Ultrasonography is a useful tool for the identification and location of metastasis in the neck; however, for lymph nodes without typical signs of metastasis (e.g., microcalcification, cystic changes) [32], it is difficult to make a decision regarding surgery through ultrasonography alone. NONHSAT076754 is of great value for the indication of the existence of LNM in PTC. Using ultrasonography and NONHSAT076754 together would reduce the rate of missed diagnoses.

Recently, accumulating evidence suggests that lncRNAs are deeply involved in cellular activities such as cell growth, proliferation, and apoptosis as well as migration and invasion through the regulation of specific genes, RNAs or proteins. Lnc-CC3 was reported to increase cervical cancer metastasis through the upregulation of Slug expression [33]. The lncRNA GClnc1, which has been functionally and clinically demonstrated to be a novel oncogene, promotes gastric carcinogenesis and may act as a modular scaffold for WDR5 and KAT2A complexes to specify the histone modification pattern [34]. It was also reported that the upregulation of the long non-coding RNA AGAP2-AS1 facilitates cell proliferation, migration and invasion, and inhibits cell apoptosis; it also represses LATS2 and KLF2 expression through its interaction with EZH2 and LSD1 in non-small-cell lung cancer cells [35]. Our study illustrated the critical function of the lncRNA NONHSAT076754 in PTC cell migration and invasion, even though this lncRNA does not influence the viability, cell cycle status or apoptosis of PTC cells. Our result indicated that NONHSAT076754 plays a critical role in PTC metastasis and that it promotes PTC progression, although the mechanism of how NONHSAT076754 regulates PTC metastasis has not been elucidated.

In conclusion, NONHSAT076754, which is associated with LNM in PTC, promotes migration and invasiveness in PTC. The addition of NONHSAT076754 led to an increase in the diagnostic value of ultrasonography. Therefore, NONHSAT076754 is a valuable auxiliary diagnostic biomarker that can be used along with ultrasonography for the prediction of cervical LNM in PTC.

## SUBJECTS AND METHODS

### Ultrasound examination

**Figure 5:**
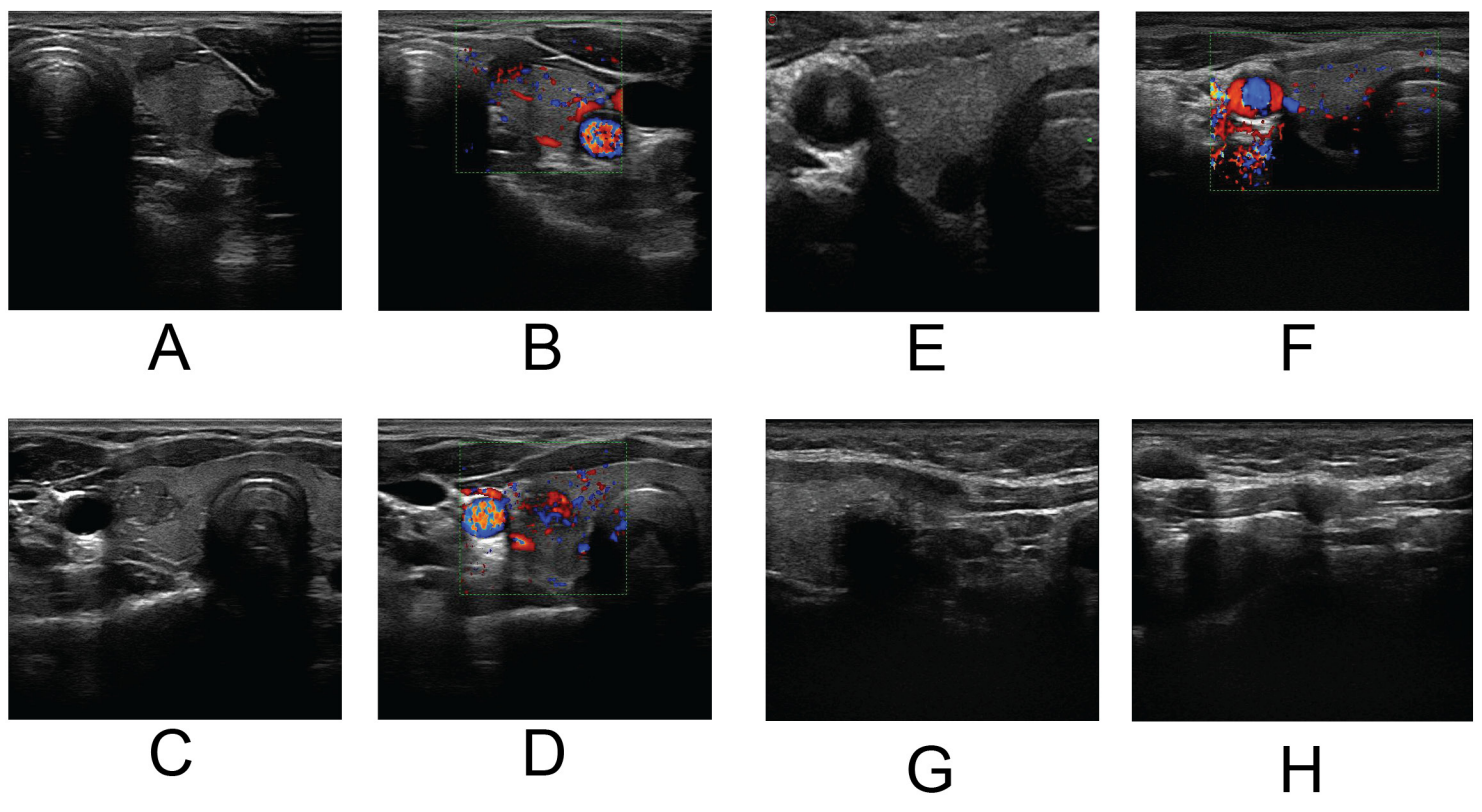
**A.&B.** A hypoechoic tumor on the left lobe with an irregular margin and 25~50% capsular abutment. Color-Doppler showed central vascularity with medium blood flow. LNM in the left central compartment was detected by ultrasonography while pathology showed no cervical LNM. **C.&D.** An isoechoic tumor on the right lobe with an irregular margin and microcalcification. Color-Doppler showed mixed vascularity with high blood flow. LNM in both left and right central compartment was detected by ultrasonography while LNM only on the right side was confirmed by pathology. **E.&F.** A markedly hypoechoic tumor on the right lobe with an irregular margin and low blood flow. No suspected LNM was detected by ultrasonography while LNM in the right central compartment were diagnosed by pathology. **G.&H.** A hypoechoic tumor on the right lobe with an irregular margin. LNM in the right central compartment was detected by ultrasonography while pathology confirmed LNM in both the left and right central compartments.

Ultrasound examination of the thyroid was performed with a 5-12 MHz linear array probe. Philips iU22 (Philips Ultrasound, USA) and ESAOTE My Lab 90 (ESAOTE, Italy) ultrasound diagnostic equipment were used for the analysis. Patients were in a supine position, and the anterior neck area was fully exposed. Transverse and longitudinal scanning of each nodule was performed by two radiologists with more than 10 years of experience in thyroid ultrasound. The ultrasonographic features of the thyroid tumors were observed in greyscale. The border (clear/obscure), margin (regular/irregular), shape, peripheral halo, internal architecture (solid/mostly solid), calcification (none/micro/macro), echogenicity, echo homogeneity, lateral shadow (presence/absence), and the degree of capsular contact were recorded. Color-Doppler ultrasound was also included to analyze the blood flow (none/ low/medium/high) and vascularity (none/circular/central/mix) of the nodules. In patients with multifocal tumors, the most suspicious nodule was analyzed. (Figure 5)

### Sample collection

Seventy-two patients who underwent thyroid surgery at Rui Jin Hospital from Sept. 2014 ~ Mar. 2015 because of papillary thyroid carcinoma were enrolled in this study. All these patients underwent preoperative ultrasound examination and ultrasound-guided fine needle aspiration and were pathologically diagnosed with papillary thyroid carcinoma with or without cervical lymph node metastasis. All of the patients underwent subtotal or total thyroidectomy with regional dissection, and none received chemotherapy or radiotherapy before surgery. Specimens and patient characteristics were collected. Tumor tissues were immediately frozen in liquid nitrogen and stored at -80°C. This study was approved by the Research Ethics Committee of our hospital, and written informed consent was obtained from all patients.

### Cell culture

The human PTC cell lines TPC1 and K1 were kind gifts from the Key Laboratory for Endocrine and Metabolic Diseases of the Chinese Health Ministry (Shanghai, China). K1 is a well-differentiated PTC cell line with metastatic properties while TPC1 is a PTC cell line that does not exhibit metastatic properties [36]. TPC1 cells were cultured in RPMI 1640 medium supplemented with HEPES (Gibco, Carlsbad, CA, USA), 10% of FBS (Gibco), 100 U/mL penicillin, and 100 μg/mL streptomycin. K1 cells were cultured in DMEM (Gibco), MCDB (Sigma, Saint Louis, Missouri, USA), and F12 (Gibco) (2:1:1) medium supplemented with 10% of FBS (Gibco), 100 U/mL penicillin, and 100 μg/mL streptomycin. Cell lines were incubated at 37°C in a humidified atmosphere of 5% CO2.

### Total RNA extraction and RT-qPCR

Total RNA was extracted from the frozen tissues using TRIzol reagent (Invitrogen, Carlsbad, CA, USA) according to the manufacturer's protocol. One microgram of total RNA was reverse transcribed into first-strand cDNA according to the protocol of the Reverse Transcription Kit (Takara, Dalian, China). RT-qPCR was performed with a SYBR® Premix Ex Taq™ II Kit (Takara, Dalian, China) according to the instructions in the VIIA7 system (Applied Biosystems, Foster City, CA, USA). GAPDH was used as an endogenous control for lncRNA in all amplification reactions. The primer sequences of NONHSAT076754 and GAPDH are as follows: NONHSAT076754, 5’-AAGTTTCTCACTCACCCACCTG-3’ (forward), 5’- GAAGCATGTACAGTTCAGCATGTG-3’ (reverse); GAPDH, 5’- AAGGTGAAGGTCGGAGTCAAC-3’ (forward), 5’- GGGGTCATTGATGGCAACAATA-3’ (reverse). Data were analyzed using the comparative Ct (2-△△Ct) method, and the value of NONHSAT076754 expression was normalized to the expression of the endogenous control. Each sample was analyzed in triplicate.

### Fluorescence in situ hybridization (FISH) and microscopic imaging

The TPC1 and K1 cell lines were incubated at 37°C in a glass bottom cell culture dish (Nest Biotechnology, Wuxi, China) until they reached 60% confluence. A Cy3-fluorescence-conjugated NONHSAT076754 probe was used for RNA-FISH. A Fluorescence in Situ Hybridization Kit was purchased from RiboBio Company (Guangzhou, China). FISH was performed according to the manufacturer's protocol, and nuclei were labeled with Hoechst33342 (Thermo Fisher Scientific, Waltham, MA, USA). Fluorescence images were obtained by a laser scanning confocal microscope (LSCM) (Carl Zeiss, Oberkochen, Germany).

### Cell line transfection

NONHSAT076754 was cloned into a pLVX-IRES-Puro expression vector (BD Biosciences, Franklin Lakes, NJ, USA). TPC1 cells were infected with the viral suspension 24 h before the start of the assay. We obtained stably transfected clones by Puromycin (Promega, 2 μg/mL) selection. A stable transfectant of the pLVX-IRES-Puro empty vector was used as a control. The expression of NONHSAT076754 was silenced by an antisense oligonucleotide (the antisense oligonucleotide sequence is 5’-TGATGTGGTGGTCTGGTCTC-3’ while the sequence of the negative control is 5’-TCTGCTCACTTGCATGCCTT-3’). The oligonucleotides were transfected into the cells at a concentration of 100 nM using Lipofectamine™2000 (Thermo Fisher Scientific, Waltham, MA, USA) according to the manufacturer's instructions.

### Scratch assay

The cells were incubated until they were 100% confluent and were then scratched with a pipet tip. The medium was replaced with fresh serum-free medium. The wound closing procedure was observed for 24 h, and images were obtained.

### Transwell assay

Transwell chambers (Costar, Corning, NY, USA) were used in the migration and invasion assays. Matrigel (BD Biosciences, Franklin Lakes, NJ, USA) was used to coat the top side of the insert membrane used in the invasion assay. Then, 200 µL serum-free medium and 1×104 cells were added to the upper chamber, while 600 µL medium with 5% FBS was added to the lower chamber. The chambers were maintained at 37°C and 5% CO2 for 24 h. Next, the cells that had not migrated or invaded the top side of the insert membrane were removed with cotton swabs. The inserts were then fixed in methanol for 20 minutes and stained with 1% crystal violet for 30 minutes. The cells that had migrated or invaded the bottom of the membrane were calculated under a microscope and photographed. All experiments were performed in triplicate.

### Colony formation assay

After treatment for 48 h, TPC1 and K1 cells were seeded in 6-well plates at a density of 500 cells/well and cultured at 37°C in a humidified atmosphere of 5% CO2; the cultured medium was replaced every other day. After 7 days in culture, the medium was removed and the cells were washed twice with PBS. Finally, the cells were stained with crystal violet for 30 minutes at room temperature, washed again and photographed.

### CCK-8 analysis

Forty-eight hours after transfection, TPC1 and K1 cells were seeded into 96-well plates at a density of 1×104 cells/well. Cell viability was evaluated by a cell counting kit-8 assay (CCK-8; Dojindo Molecular Technologies, Japan) at 24 h, 48 h, 72 h and 96 h. The absorbance at 450 nm was measured in a TECAN infinite M200 plate reader.

### Flow cytometry

Forty-eight hours after transfection, TPC1 and K1 cells were collected for the cell cycle and apoptosis analyses. The apoptosis assay was performed with an Alexa Fluor® 488 Annexin V/Dead Cell Apoptosis Kit (Thermo Fisher Scientific, Waltham, MA, USA). The cells were suspended in 100 μL binding buffer with 5 μL Annexin v and 1 μL propidium iodide and were incubated for 15 minutes in the dark. Then, 400 μL binding buffer was added and the cells were resuspended. For the cell cycle analysis, the cells were fixed in 75% ethanol overnight at 4°C. They were then stained using a Cell Cycle and Apoptosis Analysis Kit (Beyotime Biotechnology, Jiangsu, China) according to the manufacturer's instructions. The cells were analyzed by a Gallios Flow Cytometer (Beckman Coulter, USA) to quantify the percentage of apoptotic cells and the percentage of cells in different phases of the cell cycle. All experiments were performed independently and in triplicate.

### Statistical analysis

All tests and calculations were performed with Statistical Program for Social Sciences 19.0 software (SPSS, Chicago, IL, USA) and GraphPad Prism 5.0 (GraphPad Software, LaJolla, CA, USA). Descriptive variables are presented as the mean±SD. Categorical variables are described as proportions. Pearson's chi-square (X2) test was applied to examine the correlation of the NONHSAT076754 expression level with clinicopathological and ultrasonographic characteristics. An independent two-sample t test was used to compare the NONHSAT076754 expression level in the two independent groups. Logistic regression was used for the univariate analysis. The significant variables in the univariate analysis were included in the multivariate analysis. Sensitivity, specificity, positive predictive value (PPV), negative predictive value (NPV) and the diagnostic accuracy of NONHSAT076754 were assessed by ROC curve and the AUC. P < 0.05 was considered statistically significant.

## SUPPLEMENTARY MATERIALS FIGURES AND TABLES


