# Supplementary material for: NONHSAT076754 aids ultrasonography in predicting lymph node metastasis and promotes migration and invasion of papillary thyroid cancer cells
Source: Oncotarget. 2016 Nov 30;8(2):2293–306. doi: 10.18632/oncotarget.13725 (PMC5356800; doi:10.18632/oncotarget.13725)
Supplement: Supplementary file 1 [file oncotarget-08-2293-s001.pdf]

NONHSAT076754 aids ultrasonography in predicting lymph node metastasis and promotes migration and invasion of papillary thyroid cancer cells

Supplementary Material

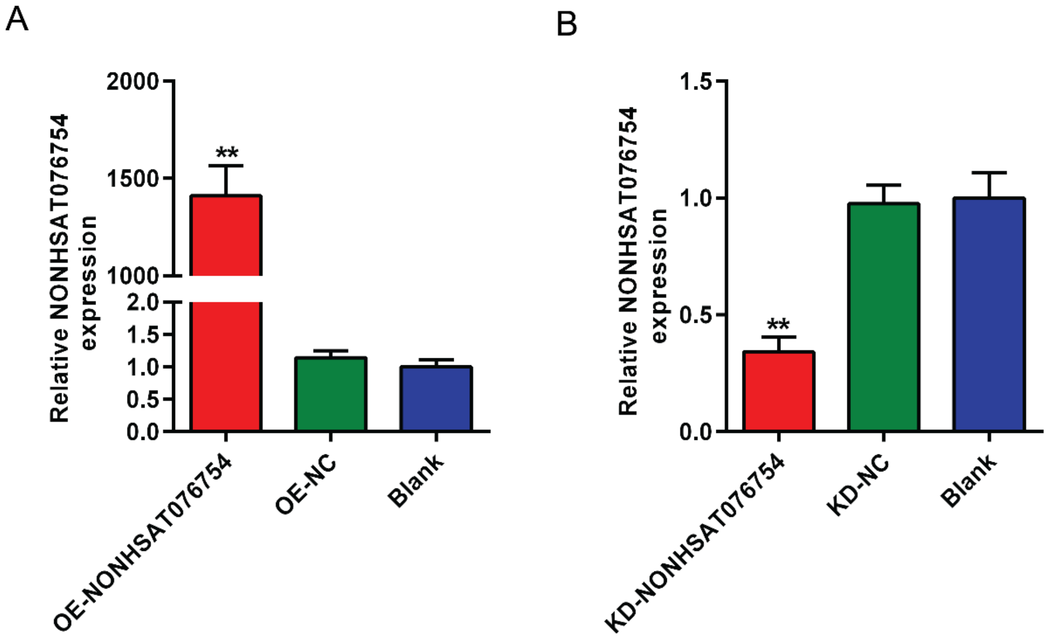

Fig. S1 The efficiency of the overexpression and knockdown of NONHSA076754 in TPC1 and K1 cells. (A) qRT-PCR analysis of the expression levels of NONHSAT076754 in TPC1 cells transfected with pLVX-NONHSAT076754 via a lentiviral vector. (B) qRT-PCR analysis of the expression levels of NONHSAT076754 in K1 cells transfected with antisense oligonucleotides by Lipofectamine. Data are expressed as the means ± SD of three independent experiments. "**" indicates P<0.01.

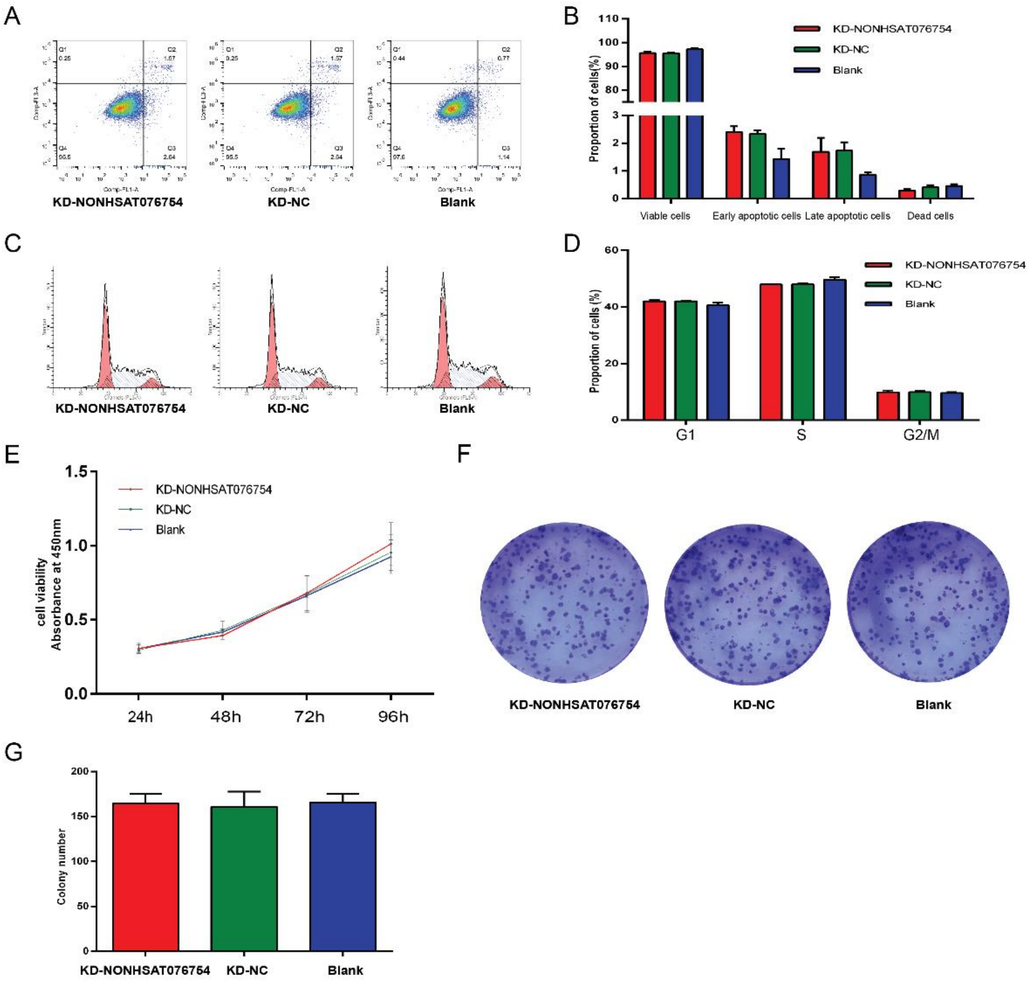

Fig. S2 Effect of NONHSAT076754 on the proliferation and cell cycle status of K1 cells. (A) Images from the flow cytometric analysis of apoptosis of K1 cells. (B) The percentage of apoptotic cells is presented in the histogram. Data are expressed as the means ± SD of three independent experiments. (C) Images from the flow cytometric analysis of the cell cycle. (D) Results quantitated in the cell cycle analysis are shown as a percentage of the total number of cells. Data are expressed as the means ± SD of three independent experiments. (E) CCK-8 assay was performed to determine the proliferation of K1 cells transfected with NONHSAT076754 after 24 hours, 48 hours, 72 hours and 96 hours. (F) A colony formation assay was performed to determine the proliferation of TPC1 cells transfected with NONHSAT076754. The colonies were captured and counted. (G) The colony formation assay results are presented in the histogram. Experiments were performed independently in triplicate, and the results are presented as the means ± SD.
